# Erector spinae plane block for cancer pain relief: a systematic review

**DOI:** 10.1186/s44158-024-00213-y

**Published:** 2024-11-15

**Authors:** Paolo Capuano, Antonietta Alongi, Gaetano Burgio, Gennaro Martucci, Antonio Arcadipane, Andrea Cortegiani

**Affiliations:** 1 Department of Anesthesia and Intensive Care, IRCCS-ISMETT, UPMC, Via Ernesto Tricomi 5, Palermo, 90127 Italy; 2 Department of Anaesthesia, Intensive Care and Emergency, University Hospital Policlinico Paolo Giaccone, Palermo, Italy; 3 Department of Precision Medicine in Medical, Surgical and Critical Care (Me.Pre.C.C.), University of Palermo, Palermo, Italy

**Keywords:** Cancer pain, Fascial plane blocks, Chronic pain, Pain managment, Locoregional anesthesia

## Abstract

**Background:**

Despite advances in pain management, cancer-related pain remains a critical issue for many patients. In recent years, there has been a growing interest in the use of fascial plane blocks, such as the Erector Spinae Plane Block (ESPB), for managing chronic pain, including in the oncology field. We conducted a systematic review to synthetize existing evidence on the use of ESPB for cancer pain management.

**Methods:**

We selected studies published between January 2016 to April 2024. A systematic search in Pubmed and Embase databases was performed. The search strategy included the following keywords and/or MeSH terms according to the controlled vocabulary of the databases sought: ((erector spinae plane block) OR (ESP block) OR (ESPB) AND ((cancer pain). We considered eligible Randomized, nonrandomized studies, case series and case reports reporting data on the use of ESPB in patients with cancer pain.

**Results:**

The search revealed 34 studies. Among these, we found one RCT, three retrospective studies, two case series, and 28 case reports for a total of 135 patients. Studies included described the use of ESPB for the management of various types of cancer pain across different conditions, including chronic thoracic cancer-related pain, abdominal visceral pain and pain related to bone metastases. Single-shot ESPB was performed in 26 studies while continuous ESPB and the use of a peripheral nerve catheter for continuous analgesia were described in 8 studies. Neurolytic ESPB was performed in 6 studies for a total of 10 patients There was a high clinical heterogeneity in terms of technique, drugs, and use of adjuvants. The lack of comparators was a major flaw, together with the low level of evidence in the majority of the included studies.

**Conclusions:**

The evidence supporting the use of ESPB for cancer pain management is currently scarce, heterogeneous, and of low quality. To better understand its potential and provide robust clinical guidance, future research needs to focus on rigorous comparative studies, standardization of techniques and larger sample sizes.

## Background

Pain is a prevalent and significant symptom in cancer patients, presenting a substantial burden for both the patients and the healthcare systems. It profoundly affects the patients’ quality of life and is responsible for thousands of emergency department (ED) visits each year, placing a considerable strain on healthcare resources [1, 2].

Even though opioids remain the cornerstone of cancer pain management, they have several limitations and about a quarter of cancer patients fail to attain sufficient pain control or experience severe side effects despite personalized analgesic therapy [1]. These limitations underscore the need for alternative pain management strategies. Interventional procedures targeting the central and peripheral nervous systems offer an alternative for managing cancer pain, but the current evidence for their efficacy is limited. Many of these techniques, such as pulsed radiofrequency, spinal cord stimulation, and intrathecal injection of local anesthetics, steroids, and other medications, are invasive. They require specialized expertise and carry the risk of serious complications [3].

**Fig. 1:**
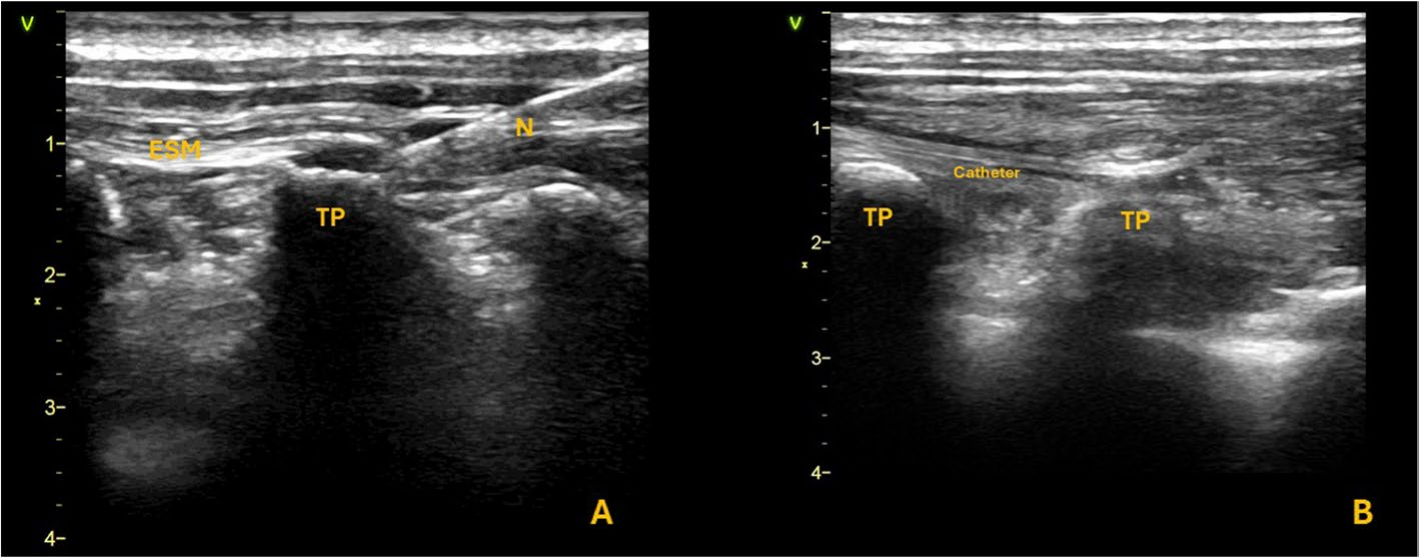
**A** Sono-anatomy of the ESPB block. The hyperechoic transverse process (TP) is individuated inferior to the erector spinae muscle. **B** Catheter placement and ultrasound visualization between the ESM and the transverse process

Over the last years, fascial plane blocks, such as the erector spinae plane block (ESPB), have emerged as valuable tools in the management of chronic pain and potentially represent a promising advancement in cancer pain management, offering a less invasive option with a strong safety profile and the potential to significantly improve pain control while reducing the need for opioids [4]. The ESPB was first described by Forero in 2016 and is considered a relatively recent addition to the field of regional anesthesia and pain management [5]. The ESPB involves the injection of local anesthetic into the interfascial plane between the erector spinae muscles and the tip of the transverse vertebral process (Fig. 1A, B).

Since its initial description, the ESPB has gained significant attention and sparked an interesting debate over its mechanism of action. Several cadaveric studies and magnetic resonance imaging have shown that the ESPB provides both somatic (related to the body wall and musculoskeletal structures) and visceral (related to internal organs) analgesia. This may explain its effectiveness in managing pain from various sources [6]. It has been observed that the local anesthetic (LA) injected into the fascial plane between the erector spinae muscles and the transverse vertebral processes can spread through channels in the intertransverse connective tissues. This spread reaches the ventral and dorsal rami of the thoracic spinal nerve, as well as the sympathetic ramus communicans at the intervertebral foramen level. Additionally, the involvement of lateral cutaneous branches of intercostal nerves enhances the block’s analgesic effect [7]. The distribution of the erector spinae muscle from the neck to the lumbar region makes the ESPB a versatile regional anesthesia technique. Its ability to provide analgesia across a wide area of the torso, including the thoracic region, has significantly contributed to its popularity in various surgical procedures [6]. This widespread applicability allows the ESPB to be used effectively in diverse clinical scenarios, enhancing its utility in both perioperative pain management and chronic pain conditions, such as those associated with cancer [4].

Multiple studies have investigated the effectiveness of ESPB for cancer-related pain. However, the specific types and doses of drugs used in ESPB, as well as their safety in the context of cancer pain, remain undetermined. To address this, we conducted a systematic review of the literature to synthetize existing evidence on the use of ESPB for cancer pain management.

## Methods

### Data source and search strategy

This systematic review was conducted according to the recommendations of Preferred Reporting Items for Systematic Reviews and Meta-Analyses (PRISMA) [8]. It was prospectively registered with the International Prospective Register of systematic reviews (PROSPERO—number CRD 42024574118).

### Search and selection strategy

Two independent reviewers (P.C. and G.M.) conducted a systematic review of the PubMed and Embase databases. The search strategy included the following keywords and/or MeSH terms according to the controlled vocabulary of the databases sought: ((erector spinae plane block) OR (ESP block) OR (ESPB) AND ((cancer pain). Publications were retrieved from January 2016 to April 2024. The two independent reviewers (P.C., G.M.) selected the studies for inclusion. Any discrepancies regarding the relevance of the papers to include would be resolved through discussion and consensus with an additional author (G.B). However, there was agreement in the selection of papers between the two authors.

Eligible studies were randomized trials, observational studies, case reports and case series in adult patients in which the ESPB (intervention) single shot, continuous, neurolytic, or non-neurolytic was used for the treatment of chronic cancer pain (population), assessed according to standard scales such as the visual analog scale (VAS), the numeric rating scale (NRS), and other (outcome), with or without comparison with other analgesic techniques or strategies (comparator). Studies describing the use of ESPB for chronic non-cancer pain, surgical and post-surgical pain, or in pediatric patients were excluded, as well as animal or cadaveric studies or studies in non-English language. We also used a snowballing method, checking the bibliographic references or citations of included studies. From each study, the following data were obtained: author name, year of publication, type of study, number of patients, type of cancer, site of cancer-related pain, ESPB technique, levels of the blockade, drugs used (percentage and volume), pain intensity before and post ESPB, and complications related to the block. The quality of evidence was evaluated using the Oxford Centre for Evidence-Based Medicine (OCEBM) 2011 Levels of Evidence methodology [9]

### Evidence synthesis and results

**Fig. 2:**
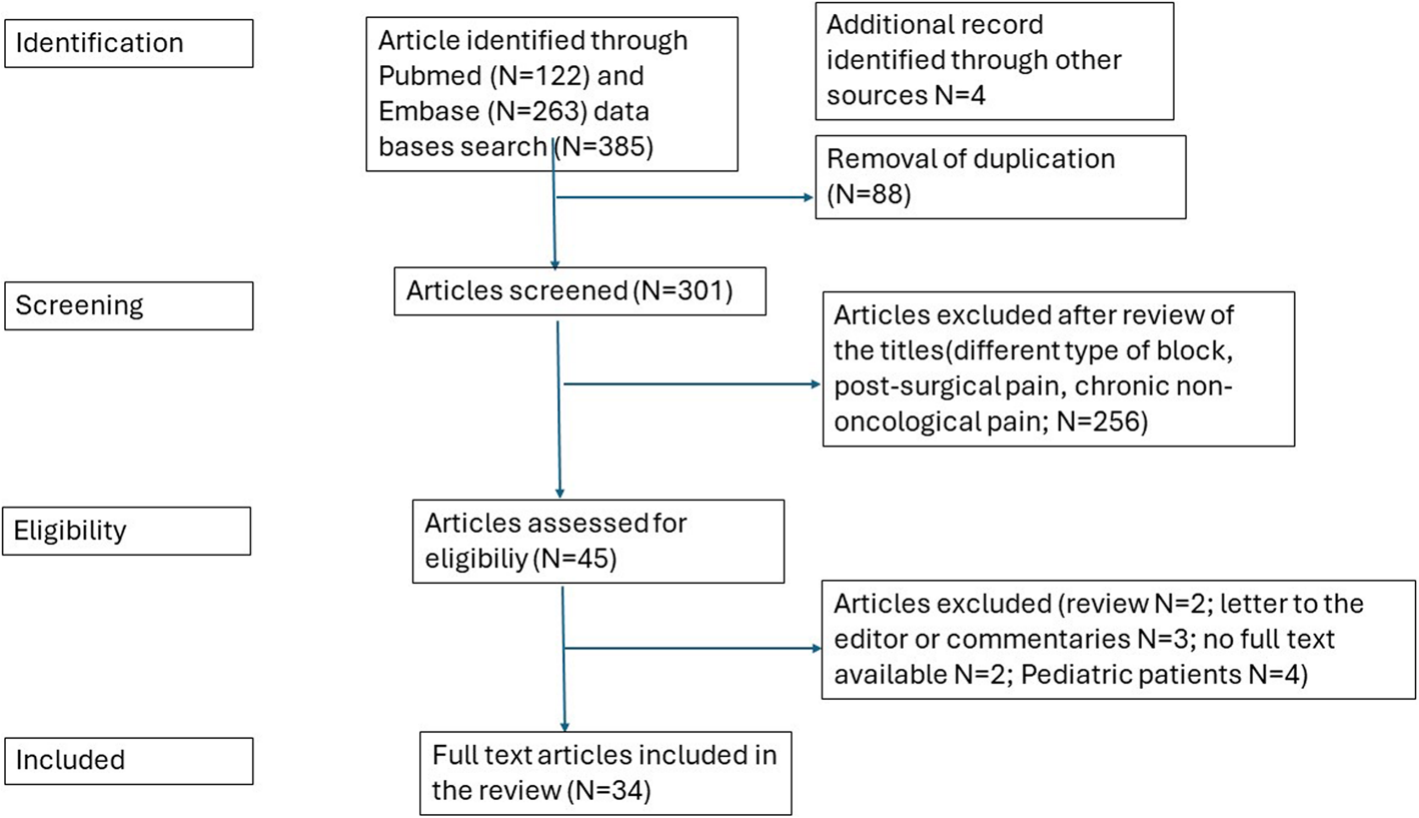
PRISMA flow diagram of the study selection and screening process

The initial search revealed a total of 389 publications. After duplicate removal, 301 articles were eligible for screening from title and abstract and 256 were excluded. The remaining 45 articles were then eligible for full-text review. Among these, 11 studies were excluded (review *N* = 2, letter to the editor or commentaries *N* = 3, no full text available *N* = 2, and pediatric patients *N* = 4). The inclusion/exclusion process is reported in Fig. 2.

The overview and main findings of the included studies are summarized in Table 1. According to OCEBM, level 2 of evidence was assigned to the only randomized trial included in this review [10], two retrospective studies were classified as level 3 [11, 12] and a retrospective study [13] (including two subgroup patients), two case series [14, 15], and all the case reports [2, 5, 16–40] were classified as level 4 of evidence. Due to the low level of evidence of included studies and the clinical heterogeneity, a meta-analysis was not feasible, and we conducted a qualitative review of the studies.

**Table 1:** Baseline characteristics of studies included in the review

**Author**	**Study design**	**Numbers of patients**	**Site of cancer pain**	**ESPB level**	**Drug used**	**Bolus/catheter**	**Preblock VAS/NRS**	**Post block VAS/NRS**	**Complications**
**Messeha MM **[10]** (2021)**	Randomized trial	30	Thoracic pain (Breast cancer (*n*=21) Chondrosarcoma (*n*=4) Osteosarcoma (*n*=3) Ewing sarcoma (*n*=2))	T1–T5	T5 Group 1: 40 mg methylprednisolone + 10 mL lidocaine 2% Group 2: 40 mg methylprednisolone + 10 mL of 2% lidocaine +50 IU of calcitonin	Single shot	6 (5-8)^a^	Group 1: 2 (1-3) at 1 week; 3 (2-6) at 2 weeks^a^ Group 2: 2 (1-4) at 1 week; 3 (2-5) at 2 weeks^a^	
**Hochberg U **[11]** (2022)**	Retrospective	44	Thoracic cancer pain	Thoracic	Unilateral: 10 mL of 1% lidocaine + dexamethasone 10 mg Bilateral: 15–20 mL of 1% lidocaine + dexamethasone 10 mg	Single shot	7.1 ± 1.3^b^	5.0 ± 2.3^b^	Injection Site soreness
**Castillo Ramirez L **[12] **(2024)**	Retrospective	16	Vertebral oncologic fractures	T1 to L4 (*n*=11 ultrasound; *n*=1 tomography; *n*=4 fluoroscopy)	Methylpredisolone 20-40 mg + Ropivacaine 0.2 % (*n*=12) Phenol 6-8 % (*n*=3) Methylpredisolone 20 mg + Bupivacaine 0.25 % (*n*=1)	Single shot	5.3 ±2.1^b^	2.7±1.9^b^	
**Ünal Artık HA **[13]** (2022)**	Retrospective	2 (subgroup)	Thoracic pain (Endometrial cancer)	T8	18 mL of bupivacaine 0.5% +8 mg dexamethasone	Single shot	8	3	
			Thoracic pain ( Over malignancy)	T9			9	3	
**Takimoto K **[14]** (2020)**	Case series	3	right flank pain (stage 4 esophageal cancer)	T10	10 mL of 0.4% levobupivacaine And 4 days later a second ESP block was performed with 10 mL of 0.25% levobupi	Single shot	10	0(16 days)	
			Back pain (stage 4 lung cancer)	T7 bilateral	10 mL of 0.4% levobupivacaine+5 mL of 0.25% levobupivacaine 6 times within the next 2 weeks	Single shot	>7	0	
			Lower abdominal pain and back pain (stage 4 urachal carcinoma)		10 mL of 0.4% levobupivacaine+10 mL of 0.25% levobupivacaine 10 days later	Single shot	10	0	
**Rispoli L **[15] **(2019**	Case series	5	Thoracic pain (Acute myeloid leukemia, renal cell carcinoma, colon cancer, prostate cancer, chordoma)	T9-T12	Bupivacaine 0.25% 6–10 mL and methylprednisolone 40–80 mg	Single shot	6-8	<4 (one patient did not respond)	
**Ashworth H **[2]** (2022)**	Case report	1	Right sided abdominal (metastatic colon cancer)	Thoracic T9	20 mL of 0.5% bupivicaine	Single shot	NA	1	
**Hernández‑Porras BC**[16] **(2020)**	Case report	1	Left hemitorax (pleural metastatic lesions from Carcinoma of the tongue)	Thoracic T5	20 mL of bupivacaine 0.25% + methylpred 40 mg	Single shot	10	0 (1 month)	Burning sensation at injection site after 12 h, hypoesthesia from left‑side T5–T12
				US and CT image	12 mL of 6% phenol	Single shot	8	2 (4 months)	
**Fusco P **[17]** (2021)**	Case report	1	Vertebral metastatis D7-D9 (parotid carcinoma)	T9	20 mL of 0.2% ropivacaine and dexamethasone 8 mg	Single shot	8	0 (4 at 1 week)	
**Kalagara HK **[18]** (2019)**	Case report	1	Right neck and thorax (pancost tumor)	T1	15 mL of 0.5% ropivacaine + 0.2% ropivacaine, 5 mL/h	Cathether (removed at day 9)	10	4	
**Altiparmak B **[19]** (2019)**	Case Report	2	Bone metastatis at T4-T5 and T3-T7	T3 and T6 bilateral	10 mL of 0.25% bupivacaine, 2 mg of dexamethasone	Bilevel Single shot	8/9	<2	
**Papa P **[20]** (2020)**	Case report	1	left supraclavicular pain (lung carcinoma)	T2	15 mL of 8% phenol	Single shot	9	0 (4 weeks)	
**Kadam VR **[21]** (2018)**	Case report	1	lung adenocarcinoma causing posterior chest wall pain	T11 (US and Fluoroscopic)	20 mL of ropivacaine 1%+11.4 mg of betamethasone	Single shot	10	0	
**Subramanian VV **[22]** (2021)**	Case report	1	Left chest wall (Carcinoma Breast)	T4	20 mL of 0.5% ropivacaine +0.2% ropivacaine, 10 mL/h	Catheter (discharged at home with catheter)	10	1	
**Sirohiya P **[23]** (2020)**	Case Report	2	Right flank (Desmoid tumor)	L2	20 mL of 0.375% ropivacaine +40 mg triamcinolone	Single shot	10	3	
			Right hemitorax (Pancoast tumor)	T2			7	2	
**Bugada D** [24]** (2020)**	Case Report	1	Abdominal cancer pain (recurrence of colorectal cancer)	T7 bilateral	20 ml of ropivacaine 0.5%+ methylprednisolone 40 mg for each side	Single shot	High (not specified)	<3 for two weeks	
**Jadon A **[25]** (2019)**	Case Report	1	Local metastasis at the right side of face	T2 (US and fluoroscopic)	Single hot: 15 mL of 0.25% bupivacaine +4mg dexamethasone Infusion: 0.08% bupivacaine + fentanyl (1.66 µg/mL) at 6 mL/h	Catheter (44 days)	10	3	
**Aydin T **[26]** (2019)**	Case Report	1	Right lower chest (pancreatic cancer)	T10	25 mL of 0.25% bupivacaine	Single shot	8	2	
**Aydin T **[27]** (2018)**	Case Report	2	Right chest and back pain (metastatic lung cancer)	T2 (US and fluoroscopic)	10 ml of 0.5% bupivacaine + 10 ml of 0.25% bupivacaine 3 times per day	Catheter	9	3	
			Left chest and back pain (mesothelioma)	T6	10 ml of 0.5% bupivacaine + 10 ml of 0.25% bupivacaine 3 times per day	Cathether	7-8	1-2	
**Ramos J** [28]** (2018)**	Case report	1	Chest wall (pleural mesothelioma)	T3	20 mL of 0.5% bupivacaine + 5 mL bupivacaine 0.5% 3 times per day	Catheter	6	2	
**Rocha‑Romero A **[29]** (2021)**	Case Report	1	Left hemitorax (Breast Cancer)	T5	17 mL of 5% phenol	Single shot	9	2 (2 weeks)	
**Fusco P **[30]** 2023**	Case report	1	left dorsal region (B-cell non-Hodgkin’s lymphoma)	T1	SS at 42 °C for a total volume of 30 mL	Single shot	6	2 (two weeks)	
**Rahimzadeh P **[31]** (2023)**	Case Report	1	cervicothoracic junction (Pancoast tumor)	T2-T3 bilateral	20 cc of ropivacaine 0.2% and triamcinolone 40 mg	Single shot	NA	NA (a pain reduction up to 70 percent is described)	
					20 cc of ROPI 0.2% and 5 cc of ozone 30 mics (second session seven days later)	Single shot			
**Gopinath B **[32]** (2022)**	Case Report	1	right upper abdominal pain (cholangiocarcinoma)	T7 bilateral	30-mL mixture of 1% lidocaine (2 mg/kg), 0.5% bupivacaine (1 mg/kg), and 4 mg dexamethasone	Single shot	8	3 (4 days)	
**Aggarwal N** [33]** (2021)**	Case report	2	Left Thoracic neuropathic pain(Metastatic lung melanoma)	T5	20 mL of bupivacaine0.2% with 10 mg of dexamethasone	Single shot	10	0 (2 week)	
			Left Thoracic pain (Metastatic lung colon cancer)	T5	13 mL of 0.25% bupivacaine was with 5 mg of dexamethasone	Single shot	10	3 (1 month)	
			Right chest pain (metastatic lung cancer)	T5	5 mL of 0.25% bupivacaine and 5 mg of dexamethasone	Single shot	9	0 (two weeks)	
			Right thoracic back pain (disseminated lung carcinoid tumor)	BilateralT5	11 mL of a 22 mL 0.2% ropivacaine and 10 mg of dexamethasone on each side	Single shot	9	6 (no follow up)	
**Mukherjee V **[34]** (2021)**	Case report	1	pulmonary metastases of prostate cancer	T7	0.5% lidocaine, 0.5% bupivacaine, and 10 mg dexamethasone.	Single shot	NA	NA	
**Nailufar A** [35]** (2019)**	Case Report	1	Thoracic pain (Pulmonary bronchogenic carcinoma)	T4-T5	20cc bupivacaine 0.125% every 12 hours	Catheter	9	1-2	
**Altiparmak B **[19]** (2019)**	Case report	2	back pain related to breast cancer metastasis P	T3 and T6 bilateral	10 mL of 0.25% bupivacaine, 2 mg of dexamethasone and 5 mL of normal saline mixture	Single shot	8	<2	
			back pain related to breast cancer metastasis	T3 and T6 bilateral	10 mL of 0.25% bupivacaine, 2 mg of dexamethasone and 5 mL of normal saline mixture		9	<2	
**Pabón‑Muñoz FE **[36]** (2019)**	Case report	1	Thoracic back pain (bone metastatis of an unknown primary tumor)	T7 bilateral	10 mL of 0.5% bupivacaine +9 mL of 1% lignocaine +4 mg dexamethasone	Single shot	10	2(two weeks)	
**Forero M **[5]** (2016)**	Case report	1	Left chest pain (Metastatic lesion of ribs)	T5	20 mL of 0.25% bupivacaine	Single shot	10	0	
**Jadon A **[37]** (2019) **	Case report	1	Metastatis at the right side of face (Carcinoma Esophagus)	T2	15 mL of 0.25% bupivacaine +4mg dexamethasone Continuous: 0.08% bupivacaine + fentanyl (1.66 µg/mL) at 6 mL/h	Catheter	10	3	
**Correa J **[38]** (2020)**	Case report	2	Left hemitorax Right hemitorax (breast cancer)	Thoracic (T3) Thoracic (T4)	15 ml Levobupivacaine 0,375% with Epinephrine 1:200.000 + Metilprednisolona 40 mg + 10 mL of 8% phenol	Single shot	10	0(1 months	
					15 ml of Levobupivacaine 0,375% + Depomedrol 40 mg and then 10 ml of phenol al 8% (total volume of 25 ml)		8	2 (5 months)	
**Cardenas J **[39]** (202) **	Case Report	2	Thoracic pain (Metastatic breast cancer)	Thoracic T4	20 mL 5% phenol	Single shot	10	4; (2 weeks)	
			Thoracic pain (Metastatic lung cancer)	Thoracic T5			8	2; (3 months)	
**Ahiskalioglu A** [40] **(2019)**	Case Report	1	Thoracic pain (Left lung carcinoma)	Thoracic T5	20 mL of 0.25% bupivacaine and continuous 0.250% bupivacaine 8 mL/h	Catheter	9	1 (3 months)	

*ESPB* Erector spinae plane block, *US* Ultrasound, *VAS* Visual Analog Scale, *NRS* Numeric Rating Scale

^a^Results are presented as median (range Q25-Q75)

^b^Results are presented as mean ± standard deviation

Among the 34 included studies, we found 1 RCT [10], 3 retrospective studies [11–13], 2 case series [14, 15], and 28 case reports [2, 5, 16–40] for a total of 135 patients.

Single-shot ESPB was performed in 26 studies [39] while continuous ESPB and the use of a peripheral nerve catheter for continuous analgesia was described in 8 studies [18, 22, 25, 27, 28, 35, 37, 40]. Neurolytic ESPB was performed in 6 studies (12,16,20,29,39,40) for a total of 10 patients. In these reports, phenol was used as neurolytic agent in a variable concentration (5% to 8%) and volume (10 to 20 ml). One case report described the use of warm saline solution (42° 30 ml) for thoracic ESPB. Regarding local anesthetics, bupivacaine [5, 13, 15, 16, 19, 25–28, 32–37, 40], and ropivacaine [12, 17, 18, 21–24, 31, 33] were the most used; however, several concentrations (0.125–0.5%) and volume (10–30 ml) were described. Twenty studies reported the use of adjuvants (calcitonin, dexamethasone, betamethasone methylprednisolone, triamcinolone, fentanyl) in the local anesthetics mixture.

No major adverse effects (bleeding, hematoma, pneumothorax, or local anesthetic toxicity) related to blocks were observed; Hochberg et al. in their retrospective analysis reported injection site soreness as the main adverse effect (without reporting its incidence).

Hernandez-Porras et al. [16] described hypoesthesia from left side T5–T12 after neurolytic ESPB with phenol 6% in a patient with pleural metastatic lesions.

## Discussion

The main finding of this systematic review is that evidence evaluating the use of ESPB is scarce, consisting of 1 small RCT, 3 retrospective studies, 2 case series, and 28 case reports. There was a high clinical heterogeneity in terms of technique, drugs, and use of adjuvants. The lack of comparators is a major flaw. This strongly limits the possibility of drawing conclusions on the effectiveness and the safety of this technique in this patient population. Of note, the total number of patients from included studies is impressively low, leading the evidence about ESBP for cancer pain relief to be considered hypothesis generating.

Only one randomized trial was included in this review: in their trial, Messeah et al. compared the effect of adding calcitonin to methylprednisolone versus methylprednisolone alone to local anesthetic in erector spinae plane block for patients suffering from thoracic cancer pain [10]. Both groups showed a decline in the VAS scores and the total tramadol consumption in the first 2 weeks after treatment and the calcitonin group showed statistically significant lower VAS values after 1, 2, and 3 months as compared to the methylprednisolone group. Also Hochberg et al., in a retrospective study, reported the benefit of ESPB for cancer-related chronic pain: in a sub-group including all patients with an active neoplastic disease-causing intractable pain refractory to medical therapy, the ESPB was effective in reducing the pain in 48% of the patients [11]. Similar results were reported by Castillo Ramirez et al. [12] in another retrospective study: they described the effectiveness of ESPB in the treatment of pain in 16 patients with vertebral oncological fractures. They reported a significant reduction in pain and oral opioid equivalent dosage in milligrams of morphine per day (MME/day) after ESP block (*p* ≤ 0.05).

Apart from the studies cited earlier, many of the remaining articles reviewed consist of low-grade evidence, primarily composed of case reports and case series. The studies mentioned involve a highly heterogeneous population, encompassing various types of cancer pain across different conditions, including chronic thoracic cancer-related pain, abdominal visceral pain, and pain related to bone metastases.

Even if this variability underscores the versatility of the ESPB in treating pain across a wide area of the torso, offering potential relief for various types of cancer pain, the inclusion of such a heterogeneous population poses significant challenges in drawing unified conclusions regarding the overall effectiveness of ESPB as a treatment. The diverse nature of pain types and underlying conditions (each with distinct pain mechanisms) makes it difficult to standardize outcomes or determine the general efficacy of ESPB across all patient groups. The differences in response to treatment among thoracic cancer, visceral pain, and bone metastases further complicate the evaluation, necessitating more condition-specific studies to gain clearer insights into ESPB’s effectiveness in each setting. Similar considerations must be made regarding the dosage of anesthetics and adjuvants used in the included studies, as there is significant variability across the trials. Corticosteroids, particularly dexamethasone, were the most frequently reported adjuvants, yet the differences in dosage and administration methods between studies make it difficult to form consistent conclusions about its efficacy in the duration of the block. Current evidence suggests that the systemic absorption and anti-inflammatory properties of dexamethasone may play a larger role than previously acknowledged, with direct perineural effects likely being minimal [41]. Given the low to moderate quality of evidence and the inconsistency across studies, clinicians should carefully consider the balance between perineural and systemic administration when using dexamethasone as an adjuvant for fascial plane blocks.

Early incorporation of interventional pain management strategies in cancer-related pain treatment has the potential to enhance outcomes, decrease dependence on opioids, and improve the quality of life for patients [42]. Among the more common interventional techniques for managing cancer-related pain, sympathetic nerve blocks and neuraxial analgesia infusions are frequently employed, particularly for patients who experience severe or refractory pain that is not well-managed by traditional methods [43].

ESPB in the treatment of chronic cancer pain can potentially represent a valid alternative to these techniques, with the dual advantage of treating somatic and visceral pain in an outpatient setting and with a lower risk of complications, especially compared to neuraxial techniques. However, the absence of a comparator in the reported studies represents a significant limitation. This flaw means that considerations drawn about ESPB effectiveness and advantages over other pain management techniques remain largely hypothetical.

Moreover, as a limitation, we acknowledge the fact that we did not perform a formal risk of bias assessment. However, in this case, we anticipated that most of the included record would have been case reports/case series or very low-quality evidence. At the protocol stage, we decided to assess the quality of the evidence by OCEBM criteria without considering a formal risk of bias assessment for the very few articles where this would have been possible. In our opinion, regardless of the specific sources of bias, the low quality of the studies included in the present review already undermined their reliability, making further bias assessment less impactful.

### Insights for future research

As the erector spinae plane block (ESPB) becomes more popular for managing chronic cancer pain, future research is expected to concentrate on several key areas to optimize its use and efficacy. Research will aim to identify the optimal dose and volume of local anesthetics for achieving effective pain relief while minimizing side effects. Evaluating different adjuvants, such as corticosteroids, dexmedetomidine, and others, will be crucial to enhance the efficacy of the ESPB. Future research should include comparative studies to evaluate the benefits and side effects of ESPB relative to other techniques, such as neuraxial blocks, sympathetic nerve blocks, or pharmacological approaches, providing a clearer picture of its role in chronic cancer pain management and help solidify its position as an alternative option. As research on the ESPB progresses, it is crucial to complement these studies with expanded anatomical and clinical investigations of the fascia, to better understand its role in acute and chronic pain management. Initially, the fascia was primarily regarded as a container for local anesthetic, with its main role believed to be facilitating nerve blockade within the fascial plane [44]. Today, we known that fasciae are richly innervated, containing Pacini and Ruffini corpuscles, and abundant free nerve endings suggesting a critical role in proprioception, balance, and, importantly, pain perception [45].

In a case report included in this review, Fusco et al. [30] reported the administration of warm saline solution alone within the fascial plane in a patient suffering from chronic myofascial syndrome due to chemotherapy for vertebral localizations of a B cell non-Hodgkin’s lymphoma. The authors hypnotized that mechanical stimulation of the free nerve endings caused a remodulation of pain transmission, together with a breakdown of the hyaluronan macromolecules, restoring the physiological sliding to the fascia, resulting in greater freedom of the neck movements and reduction of the painful sensation immediately after the procedure, as confirmed by elastography images [30].

Although the hypothesis is indeed fascinating, further validation is necessary to confirm that injecting a larger volume of warm saline solution (SS) into the fascial plane can effectively reduce muscle stiffness and improve pain symptoms.

An innovative and certainly interesting application is represented by the use of neurolytic techniques. Neurolytic blocks, which involve the use of substances like phenol or alcohol, are advantageous because they are cost-effective, can provide long-term relief with a single administration, and can be easily repeated if the effect is short-lived.

This is particularly true for patients suffering from neuropathic pain associated with cancer, the incidence of which is estimated at around 70% [38]. Hernandez-Porras et al. reported their experience using a mixture of phenol and contrast medium in order to explore the spread of injectate through CT image [16]. Moreover, the same group proposed a step-wise approach first beginning with a diagnostic block, then a catheter infusion step, and finally the phenol neurolysis with slow phenol injection (fractional doses of 1 mL·min-1) to make the procedure even safer [29]. However, a careful risk–benefit assessment is recommendable for neurolytic techniques, particularly considering the unpredictable spread in ESPB and possible fearful consequences such as paraspinal muscle atrophy [46].

## Conclusions

The evidence evaluating ESBP in cancer pain is scarce, heterogeneous, and of low quality. The overall number of patients studied is impressively low. There is high clinical heterogeneity in terms of characteristics of the technique, drugs used, adjuvants, and outcomes. Controlled studies are needed to properly evaluate the effectiveness and side effects of ESPB in cancer pain management and provide robust evidence to guide clinical practice in this patient population.

## Data Availability

No datasets were generated or analysed during the current study.

## Abbreviations

ED: Emergency department
ESPB: Erector spinae plane block
LA: Local anesthetic
PRISMA: Preferred Reporting Items for Systematic Reviews and Meta-Analyses
VAS: Visual analogue scale
NRS: Numeric rating scale
OCEBM: Oxford Centre for Evidence-Based Medicine
MME: Milligrams of morphine
SS: Saline solution
